# A new view on the scenario of karyotypic stasis in Epinephelidae fish: Cytogenetic, historical, and biogeographic approaches

**DOI:** 10.1590/1678-4685-GMB-2021-0122

**Published:** 2021-11-15

**Authors:** Karlla Danielle Jorge Amorim, Gideão Wagner Werneck Félix da Costa, Marcelo de Bello Cioffi, Alongklod Tanomtong, Luiz Antônio Carlos Bertollo, Wagner Franco Molina

**Affiliations:** 1 Universidade Federal do Rio Grande do Norte, Departamento de Biologia Celular e Genética, Centro de Biociências, Natal, RN, Brazil.; 2 Universidade Federal de São Carlos, Departamento de Genética e Evolução, Laboratório de Citogenética de Peixes, São Carlos, SP, Brazil.; 3 Khon Kaen University, Department of Biology, Faculty of Science, Muang, Khon Kaen, Thailand.; 4 Khon Kaen University, Toxic Substances in Livestock and Aquatic Animals Research Group, Muang, Khon Kaen 40002, Thailand.

**Keywords:** Groupers, animal cytogenetics, pericentric inversions, rDNA, karyotype evolution

## Abstract

Epinephelidae (groupers) is an astonishingly diverse group of carnivorous fish widely distributed in reef environments around the world, with growing economic importance. The first chromosomal inferences suggested a conservative scenario for the family. However, to date, this has not been validated using biogeographic and phylogenetic approaches. Thus, to estimate karyotype diversification among groupers, eight species from the Atlantic and Indian oceans were investigated using conventional cytogenetic protocols and fluorescence *in situ* hybridization of repetitive sequences (rDNA, microsatellites, transposable elements). Despite the remarkable persistence of some symplesiomorphic karyotype patterns, such as all species sharing 2n=48 and most preserve a basal karyotype (2n=48 acrocentrics), the chromosomal diversification in the family revealed an unsuspected evolutionary dynamic, where about 40% of the species escape from the ancestral karyotype pattern. These karyotype changes showed a relation with the historical biogeography, likely as a byproduct of the progressive occupancy of new areas (huge diversity of adaptive and speciation conditions). In this context, oceanic regions harboring more recent clades such as those of the Indo-Pacific, exhibited a higher karyotype diversity. Therefore, the karyotype evolution of Epinephelidae fits well with the expansion and geographic contingencies of its clades, providing a more complex and diverse scenario than previously assumed.

## Introduction

Reef regions are home to a huge diversity of fish (Bezerra and Silva, 2011), among which Epinephelidae (groupers) stand out for their exceptional diversity. The family and allies (Epinephelidae and Serranidae) include 593 species and 71 genera distributed around the world (Craig and Hastings, 2007; Vaini *et al.,* 2019; Fricke *et al.,* 2021), with the greatest species richness being concentrated in the Indo-Pacific region (Bawole *et al.,* 2018). 

Groupers present a broad reproductive strategy, including synchronous and asynchronous hermaphroditism (Pressley, 1981; Liu and Sadovy, 2004). Some species can reach up to more than 400 kg (Bright *et al.,* 2016), making them an important target for commercial fishing and fish farming (Heemstra *et al.,* 2002; Rimmer and Glamuzina, 2017). Commercial exploitation has placed groupers among the marine species most impacted by commercial fishing, with 12% of species under threat of extinction (Mitcheson *et al.,* 2013). Some biological characteristics contribute to the low restoration of their populations such as slow growth, late maturation, high longevity (i.e., almost 40 years of life), and formation of large agglomerations during the reproductive period (Craig *et al.,* 2011; Santos *et al.,* 2019). However, some species such as the Atlantic goliath grouper (*Epinephelus itajara*) have responded to conservation measures (Giglio *et al.,* 2014).

Molecular approaches have better clarified the phylogenetic relationships of the family (Minglan *et al.,* 2014; Ma *et al.,* 2016; Ma and Craig, 2018; Saad, 2019). In contrast, cytotaxonomic data are still extremely limited, comprising only 8% of the group representatives. In addition, most of the available information refers to *Epinephelus* species, and is restricted to conventional analyses of the karyotype (Arai, 2011; Pinthong *et al.,* 2013; Paim *et al.,* 2017). 

Most Epinephelidae species have a karyotype composed of 2n = 48, with a predominance of acrocentric chromosomes (Arai, 2011; Tseng and Shih, 2018), suggesting the maintenance of a basal karyotype with a low evolutionary dynamic. However, chromosomal data of a larger number of representatives, considering their complex evolutionary biogeographical characteristic (Ma *et al.,* 2016; Ma and Craig, 2018), have been entirely neglected, still missing pieces for inferences on the extent of the karyotype stability in the family (Motta-Neto *et al.,* 2019). 

Thus, to understand the mechanism of karyotype evolution among Epinephelidae in depth, conventional cytogenetic analyses and chromosomal mapping of six repetitive DNA classes were performed in eight species from the Atlantic and Indian oceans. The data obtained were associated with a set of other available information, thereby providing a comprehensive view of the chromosomal evolution in a phylogenetic and geographic context.

## Material and Methods

### Samples, chromosomal preparations, and analyses

Eight species belonging to three Epinephelidae genera, *Epinephelus* Bloch, 1793: *E. itajara* (Lichtenstein, 1822), *E. adscensionis* (Osbeck, 1765), *E. coeruleopunctatus* (Bloch, 1790), *E. erythrurus* (Valenciennes, 1828), and *E. sexfasciatus* (Valenciennes, 1828); *Cephalopholis* Bloch and Schneider, 1801: *C. fulva* (Linnaeus, 1758) and *C. formosa* (Shaw, 1812); and *Rypticus* Cuvier, 1829: *R. saponaceus* (Bloch and Schneider, 1801) were analyzed. The experiments followed ethical rules approved by the Animal Ethics Committee of the Federal University of Rio Grande do Norte (Process #44/ 2015), and by the Institutional Animal Care and Use Committee of Khon Kaen University, based on the Ethics of Animal Experimentation of the National Research Council of Thailand IACUC-KKU-10/62.

**Figure 1 -:**
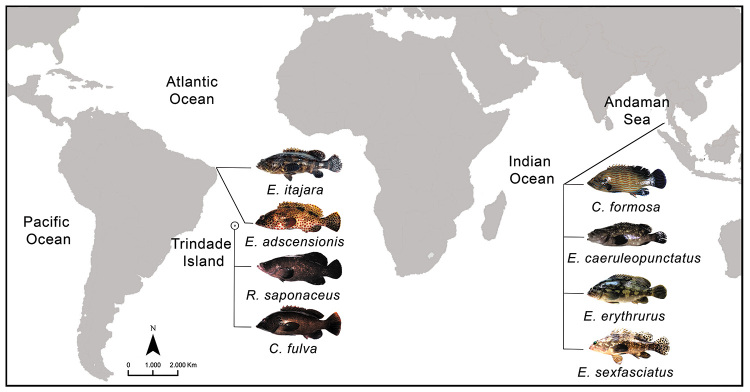
Collection sites of *Epinephelus itajara, Epinephelus adscensionis, Rypticus saponaceus,* and *Cephalopholis fulva* species, all from the Atlantic Ocean, and of *Cephalopholis formosa, Epinephelus coeruleopunctatus, Epinephelus erythrurus,* and *Epinephelus sexfasciatus* species, all from the Indian Ocean.

Details of the size and location of the samples are presented in Table 1 and Figure 1. Individuals were subjected to a 24 h mitotic stimulation using intraperitoneal inoculation of a complex of fungal and bacterial antigens (Molina *et al.,* 2010). Chromosome preparations were obtained from cell suspensions of the anterior region of the kidney using a short-term culture as described by Gold *et al.* (1990). Chromosomes were stained using a standard 5% Giemsa solution (pH 6.8) and analyzed under an optical microscope at a magnification of 1000×. The nucleolus organizing regions (NORs) and C-positive heterochromatin were identified following Howell and Black (1980) and Sumner (1972), respectively.

**Table 1 -:** Epinephelidae species analyzed in the present study.

Genera/Species	n	Collection regions	Coordinates
***Epinephelus***			
*E. itajara*	1	Rio Grande do Norte State, NE Brazil - Western Atlantic	6°19’23,38” S, 35°02’48,84” W
*E. adscensionis*	6	Rio Grande do Norte State, NE Brazil - Western Atlantic	6°19’23,38” S, 35°02’48,84” W
*E. coeruleopunctatus*	3	Andaman Sea - Thailand - Indian Ocean	11°04’00” N, 95°44’34” E
*E. erythrurus*	1	Andaman Sea - Thailand - Indian Ocean	11°04’00” N, 95°44’34” E
*E. sexfasciatus*	3	Andaman Sea - Thailand - Indian Ocean	11°04’00” N, 95°44’34” E
***Cephalopholis***			
*C. fulva*	5	Trindade Island - Brazil	20°30’38,84” S, 29°19’22,97” W
*C. formosa*	4	Andaman Sea - Thailand - Indian Ocean	11°04’00” N, 95°44’34” E
***Rypticus***			
*R. saponaceus*	4	Trindade Island - Brazil	20°30’38,84” S, 29°19’22,97” W

### Probes for chromosome hybridization

5S rDNA (~ 200 bp) and 18S rDNA (~ 1400 bp) probes were obtained by PCR from the nuclear DNA of *Rachycentron canadum* (Teleostei, Perciformes) using the primers A 5’-TAC GCC CGA TCT CGT CCG ATC-3’ and B 5’- CAG GCT GGT ATG GCC GTA AGC-3’ (Pendás *et al.,* 1994), and NS1 5’-GTA GTC ATA TGC TTG TCT C-3’ and NS8 5’-TCC GGT GCA TCA CCT ACG GA-3’ (White *et al.,* 1990), respectively. 5S rDNA and 18S rDNA probes were labeled by nick translation with biotin-14-dATP and digoxigenin-11-dUTP, respectively, according to the manufacturer’s specifications (Roche Mannheim, Germany). *Tol2* (~ 200 bp) and *Rex3* (~ 200 bp) probes were amplified using PCR from the nuclear DNA of *E. itajara* using the primers *Tol2*-5F 5′ -CTG TCA CTC TGA TGA AAC AG-3′ and *Tol2*-5R 5′ -CTT TGA CCT TAG GTT TGG GC-3′ (Kawakami and Shima, 1999) and *Rex3*-F5’ -YAA TGA CGG AGG GCC CGG CA-3′ and *Rex3*-5′-TGG GTG GTG GGG CAG GT ACN-3′ (Volff *et al.,* 1999; 2000) and labeled with digoxigenin-11-dUTP by nick translation (Roche Mannheim, Germany). *In situ* hybridizations with (CA)_15_ and (GA)_15_ microsatellites were performed as described by Kubat *et al.* (2008) using oligonucleotides labeled with Alexa Fluor 555 at the 5’ terminal position (InvitrogenTM, Thermo Fisher Scientific, California, USA). 

### Hybridization experiments

Fluorescence *in situ* hybridization (FISH) was performed as described by Pinkel *et al.* (1986). Chromosomes were treated with RNAse (20 µg/mL in 2× SSC) for 1 h and with pepsin (0.005% in 10 mM HCl) for 10 min at 37 °C, followed by a step of fixation with 1% formaldehyde for 10 min and dehydration in an alcoholic series (70%/85%/100%) for 5 min. The slides were incubated in 70% formamide/2× SSC for 5 min at 72 °C and dehydrated in an alcohol series (70%/85%/100%) for 5 min. The hybridization process was performed for 16 h at 37 °C using a hybridization solution of 50% formamide, 2× SSC, 10% dextran sulfate, and denatured probe (5 ng/µL) in a final volume of 30 µL. Post-hybridization washes were performed in 15% formamide/0.2× SSC for 20 min at 42 °C, followed by washes in 0.1× SSC for 15 min at 60 °C and in Tween-20 0.5%/4× SSC for 5 min at 25 °C. Subsequently, the slides were incubated for 15 min in 5% non-fat dry milk (NFDM)/4× SSC blocking buffer and washed in 0.5% Tween-20/4× SSC for 15 min. The hybridization signals were detected using a streptavidin-FITC conjugate for the 5S rDNA probe and anti-digoxigenin rhodamine conjugate (Roche Mannheim, Germany) for the 18S rDNA probe. Chromosomes were counterstained with Vectashield/DAPI (1.5 µg/mL) (Roche Mannheim, Germany). 

### Digital image processing

The best metaphases were photographed using an Olympus BX51 epifluorescence microscope coupled with an Olympus DP73 digital capture system using the cellSens^®^ software (Olympus). Chromosomes were defined as metacentric (*m*), submetacentric (*sm*), subtelocentric (*st*), and acrocentric (*a*), according to Levan *et al.* (1964). To count the chromosome arms (FN), the m, sm, and st chromosomes were considered with two arms and the acrocentric chromosomes with only one arm.

## Results

**Figure 2 -:**
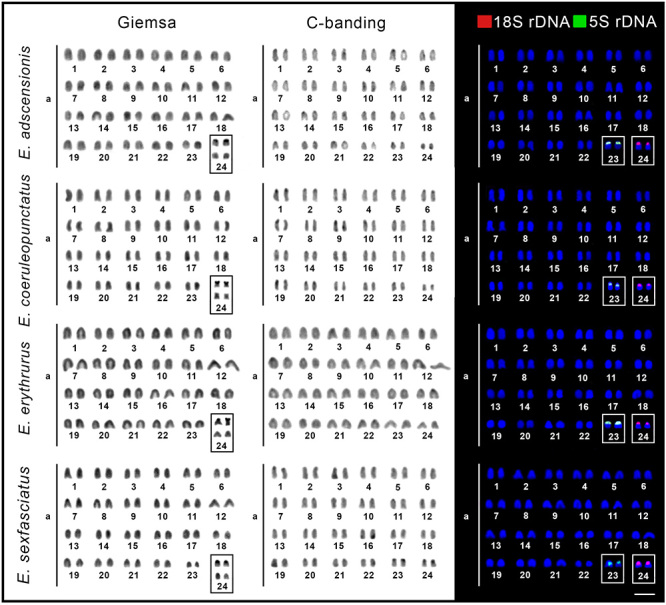
Karyotypes of *Epinephelus adscensionis, Epinephelus coeruleopunctatus, Epinephelus erythrurus,* and *Epinephelus sexfasciatus* after Giemsa staining, C-banding, and fluorescence *in situ* hybridization with 18S (red) and 5S (green) rDNA probes. Chromosomes carrying Ag-NORs sites are highlighted in the boxes. Scale bar = 5 μm.

**Figure 3 -:**
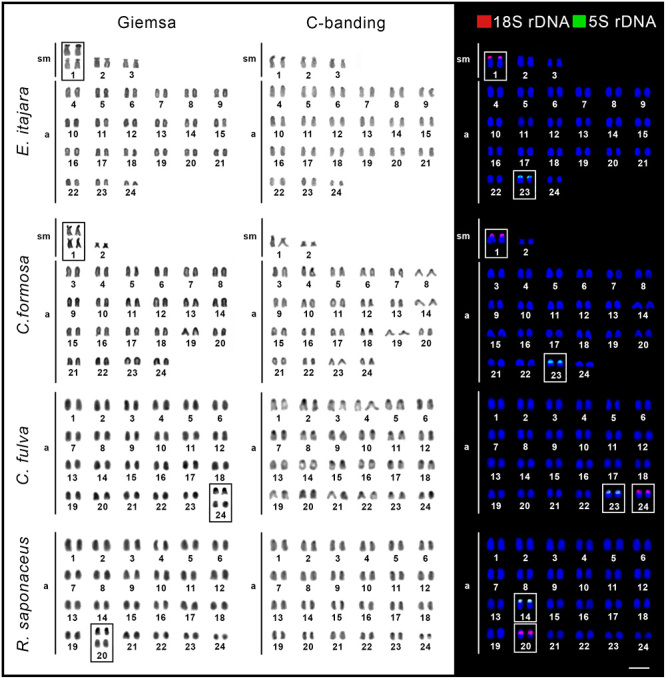
Karyotypes of *Epinephelus itajara*, *Cephalopholis formosa*, *Cephalopholis fulva*, and *Rypticus saponaceus* after Giemsa staining, C-banding, and fluorescence *in situ* hybridization with 18S (red) and 5S (green) rDNA probes. Chromosomes carrying Ag-NORs sites are highlighted in the boxes. Scale bar = 5 μm.

All analyzed species shared the same 2n = 48 chromosome number. However, while *E. adscensionis, E. coeruleopunctatus, E. erythrurus, E. sexfasciatus, C. fulva*, and *R. saponaceus* showed karyotypes composed exclusively by acrocentric chromosomes (FN = 48a), *E. itajara* had 6sm + 42a (FN = 54), and *C. formosa* had 4sm + 44a (FN = 52) chromosomes. In all species, small-sized heterochromatic blocks were localized mainly in the centromeric regions of the chromosomes (Figures 2 and 3).

The 18S rDNA and the Ag-NOR sites were coincident and occupied a single locus in the karyotype of all species, always in the short arms of the chromosomes. In *E. adscensionis, E. coeruleopunctatus, E. erythrurus, E. sexfasciatus*, and *C. fulva*, they were localized in the acrocentric pair 24 (Figure 2), while were localized in the submetacentric pair 1 of *E. itajara* and *C. formosa*, and in the acrocentric pair 20 of *R. saponaceus* (Figure 3). The 5S rDNA sequences also displayed a single site in the short arms of the chromosomes in all species. In *E. adscensionis, E. coeruleopunctatus, E. erythrurus*, *E. sexfasciatus, E. itajara*, *C. formosa*, and *C. fulva* they occurred in the acrocentric pair 23 and in the acrocentric pair 14 of *R. saponaceus* (Figures 2 and 3).

**Figure 4 -:**
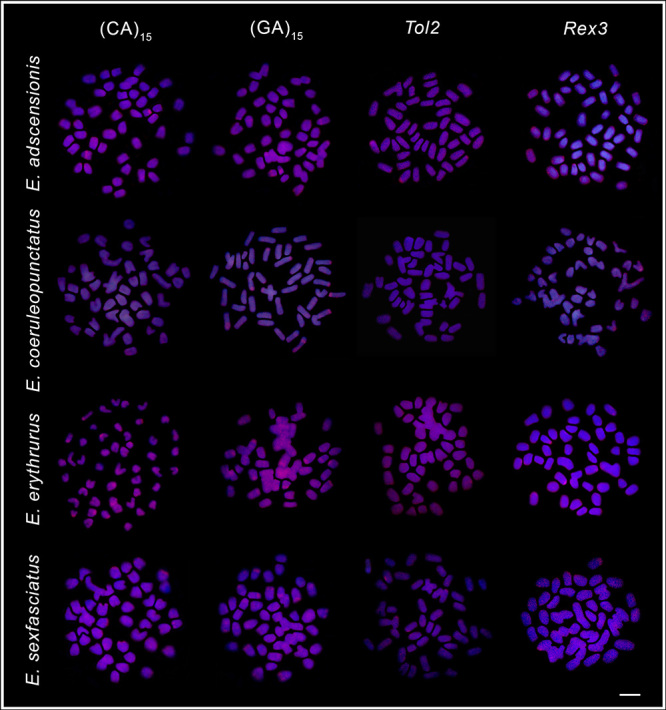
Fluorescence *in situ* hybridization mapping of (CA)_15_ and (GA)_15_ microsatellites, and *Tol2* and *Rex3* transposable elements, in mitotic chromosomes of *Epinephelus adscensionis, Epinephelus coeruleopunctatus, Epinephelus erythrurus*, and *Epinephelus sexfasciatus*. Scale bar = 5 μm.

**Figure 5 -:**
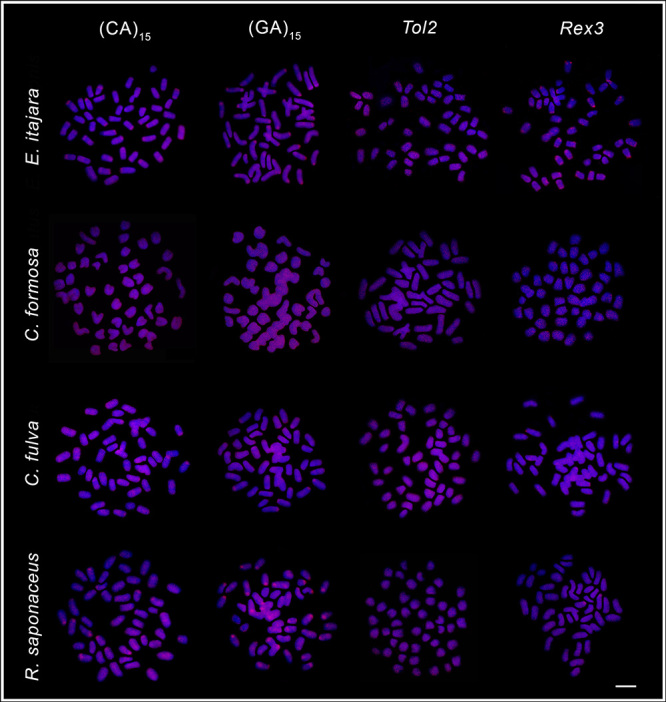
Fluorescence *in situ* hybridization mapping of (CA)_15_ and (GA)_15_ microsatellites, and *Tol2* and *Rex3* transposable elements, in mitotic chromosomes of *Epinephelus itajara, Cephalopholis formosa, Cephalopholis fulva*, and *Rypticus saponaceus*. Scale bar = 5 μm.

The microsatellites (CA)_15_ and (GA)_15_ had a scattered chromosomal distribution, with some more prominent clusters in the centromeric and terminal regions of some pairs (Figures 4 and 5). *Tol2* transposons also showed a diffuse distribution, while *Rex3* presented discrete accumulations in the centromeric and terminal chromosomal regions in all species, especially in *E. itajara*, in which more evident signals were detected (Figures 4 and 5). 

## Discussion

### Chromosomal profiles

Most Perciformes fish have retained considerable levels of chromosomal conservatism, with karyotypes composed of 2n = 48a and FN = 48 (Motta-Neto *et al.,* 2019). The distribution of such karyotype among several Epinephelidae clades (Table 2), including the ancient *Plectropomus* clade (~ 36 Mya) and recent lineages such as *Alfestes* (~ 5 Mya; Ma *et al.,* 2016), supports 2n = 48a as the basal state for this family. 

**Table 2 -:** Cytogenetic data available for groupers (Epinephelidae and Serranidae) species.

Species	2n	Karyotypes	FN	References
*Alfestes afer*	48	48a	48	Molina et al., 2002
*Cephalopholis formosa*	48	2m+46a	50	Pinthong et al., 2013; **Present study**
*C. fulva*	48	48a	48	Present study
*Centropristis ocyurus*	48	28m+20sm	96	Gonzalez and Figueras, 1990
*C. striata*	48	24m+22sm+2a	94	Merritt and Lacks, 1991
*Cromileptes altivelis*	48	2sm+ 46a	50	Takai and Ojima, 1995
*Diplectrum eumelum*	48	2m+4sm+42a	54	Aguilar and Galetti, 1997
*D. formosum*	48	2m+46a	50	Aguilar and Galetti, 1997
*D. radiale*	48	48a	48	Aguilar and Galetti, 1997
*Epinephelus adscensionis*	48	48a	48	Molina et al., 2002; **Present study**
*E. akaara*	48	48a	48	Wang et al., 2004
*E. alexandrinus*	48	48a	48	Martinez et al., 1989
*E. awoara*	48	48a	48	Wang et al., 2012
*E. bleekeri*	48	48a	48	Cai et al., 2012
*E. bruneus*	48	2m+4sm+42a	54	Minglan et al., 2014
*E. caninus*	48	48a	48	Rodríguez-daga et al. 1993
*E. coeruleopunctatus*	48	2sm+ 46a	48	Present study
*E. coioides*	48	48a	48	Wang et al., 2010
*E. diacanthus*	48	2m+46a	50	Natarajan and Subrahmanyam, 1974
*E. erythrurus*	48	48a	48	Pinthong et al., 2015; **Present study**
*E. fario*	48	4m+6sm+4st+34a	62	Zheng; et al. 2005
*E. fasciatomaculosus*	48	48a	48	Li and Peng, 1994
*E. fasciatus*	48	48a	48	Li and Peng, 1994
*E. faveatus*	48	2sm+46a	50	Magtoon and Donsakul, 2008
*E. flavocaeruleus*	48	48a	48	Tseng and Shih, 2018
*E. fuscoguttatus*	48	2sm+46a	50	Tseng and Shih, 2018
*E. guaza*	48	48a	48	Martinez et al., 1989
*E. guttatus*	48	48a	48	Medrano et al., 1988
*E. itajara*	48	6sm+42a	54	Present study
*E. lanceolatus*	48	6m+2st+40a	56	Tseng and Shih, 2018
*E. malabaricus*	48	48a	48	Zou et al., 2005
*E. marginatus*	48	48a	48	Sola et al., 2000
*E. merra*	48	4m+6sm+4st+34a	62	Zheng et al., 2005
*E. moara*	48	4sm+44a	52	Minglan et al., 2006
*E. ongus*	48	48a	48	Rishi and Haobam, 1984
*E. polyphekadion*	48	6sm+42a	54	Tseng and Shih, 2018
*E. sexfasciatus*	48	2sm+ 46a	50	Chen et al., 1990; **Present study**
*E. striatus*	48	48a	48	Amorim *et al*., unpublished data
*E. tauvina*	48	8sm+40a	56	Amorim *et al*., unpublished data
*E. tukula*	48	2sm+46t	50	Tseng and Shih, 2018
*Mycteroperca acutirostris*	48	48a	48	Aguilar, 1993
*M. rubra*	48	48a	48	Aguilar and Galetti, 1997
*Paracentropristis hepatus*	48	48a	48	Martinez et al., 1989
*Paralabrax dewegeri*	48	48a	48	Nirchio et al., 2014
*P. nebulifer*	48	48a	48	Martinez-Brown et al., 2012
*P. maculatofasciatus*	48	48a	48	Martinez-Brown et al., 2012
*Plectropomus leopardus*	48	48a	48	Pinthong et al., 2013
*Rypticus saponaceus*	48	48a	48	Present study
*R. randalli*	48	48a	48	Paim et al., 2017
*Serranus cabrilla*	48	48a	48	Martinez et al., 1989
*S. flaviventris*	48	48a	48	Aguilar and Galetti, 1997
*S. scriba*	48	48a	48	Martinez et al., 1989

The maintenance of this diploid number in all analyzed species represents a phylogenetic pattern in Epinephelidae. On the other hand, the karyotype macrostructure (2n = 48a; FN = 48), although still retained in most groupers, behaves as a more dynamic evolutionary trait. In fact, similar to *E. itajara* (2n = 48; FN = 54) and *C. formosa* (2n = 48; FN = 52), over 40% of the Epinephelidae species have some karyotype diversification associated with pericentric inversions, thereby increasing the number of chromosome arms (FN = 48-96) (Table 2). This evolutionary trend, which has been better evidenced as chromosomal data increase, is considered as a moderate diversification and reveals an unexpected context for Epinephelidae.

**Figure 6 -:**
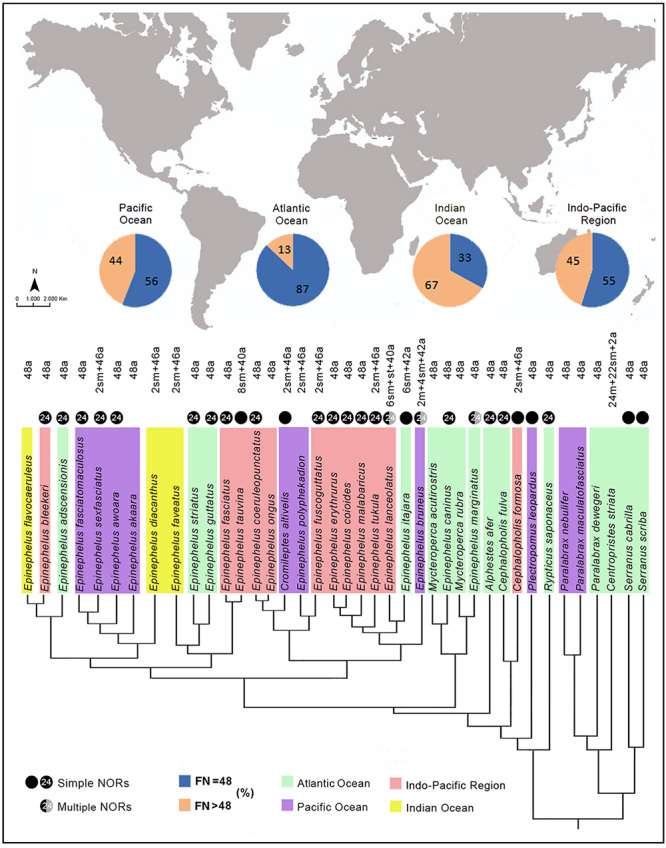
Karyotypic patterns of groupers (Epinephelidae and Serranidae) species from biogeographic and phylogenetic (based on Ma *et al.,* 2016) perspectives. The larger circles indicate the percentage of chromosome arms (FN) in the karyotypes according to the oceanic distribution of the species. Smaller black circles indicate the occurrence of a single Ag-NORs locus (24 pair or other), and the black/gray ones indicate the multiple Ag-NORs loci, according to their distribution in the chromosome pairs.

A low rate of evolutionary changes is also evidenced in some repetitive DNA sequences, as highlighted by remarkable homeologies among the Ag-NOR/18S rDNA-bearing pairs in most Epinephelidae species. Indeed, in addition to five of the eight species analyzed (*E. adscensionis, E. coeruleopunctatus, E. erythrurus, E. sexfasciatus* and *C. fulva*), the localization of the major rDNA sites on the smallest pair of the karyotype (pair 24) is a symplesiomorphic array shared by a vast number of species (e.g. Martinez *et al.,* 1989; Zou *et al.,* 2005; Wang *et al.,* 2012; Tseng and Shih, 2018), as indicated in Figure 6. In addition, non-syntenic arrays of the 18S and 5S loci, which are also frequent among teleost groups (Lucchini *et al.,* 1993; Suzuki *et al.,* 1996; Gornung, 2013), are present in all of the eight species analyzed, as well as in several other serranids (Sola *et al.,* 2000; Wang *et al.,* 2012; Paim *et al.,* 2017) (Figure 6). However, in spite of this, some alternative arrangements such as multiple 18S rDNA sites (Minglan *et al.,* 2014) or the co-localization of the 18S/5S sites in the same chromosome pair (Amorim *et al.,* unpublished data) can occur, although not expressively. The distribution of heterochromatin also offers a little discriminatory condition, since it is commonly located in the centromeric/pericentromeric regions, as observed in all the species analyzed, as well as in many other Percomorpha groups (Sola *et al.,* 2000; Motta-Neto *et al.,* 2011; Minglan *et al.,* 2014; Noikotr *et al.,* 2014).

Karyotype conservatism is thought to be related to a high level of synteny, with chromosomal sharing similar gene organization and DNA classes arrays (Ellegren, 2010; Zhang *et al.,* 2019). In this respect, the chromosomal prospecting of a diversified set of repetitive sequences allowed the estimation of evolutionary changes in different fish groups (Cioffi and Bertollo, 2012; Costa *et al.,* 2015; Lima-Filho *et al.,* 2015; Getlekha *et al.,* 2016a). In the present study, (CA)_15_ and (GA)_15_ microsatellites showed a dispersed distribution among chromosomes, with sporadic clusters in the centromeric heterochromatin of some species. This pattern contrasts with that presented by several Percomorpha species (Costa *et al.,* 2015), where conspicuous and diversified chromosomal clusters occur within the same species or among co-familiar species (Silva *et al.,* 2020).

Transposable elements, which can act at different genetic levels, including epigenetic regulation, are important components of the genome of marine fish (Aparicio *et al.,* 2002; Terencio *et al.,* 2015; Xiao *et al.,* 2020). In most of the analyzed species, *Tol2* presented a dispersed distribution in the karyotype, except for some centromeric clusters in *E. adscensionis*. In turn, *Rex3* showed a more discriminated distribution, with conspicuous accumulation in multiple centromeric and telomeric regions, mainly in *E. itajara*, a species displaying a more differentiated karyotype among the eight analyzed. This transposable element overlaps with heterochromatic regions, probably co-located with the microsatellites (CA)_15_ and (GA)_15_, which suggests a shared evolution of both repetitive DNA classes, as also proposed for other fish species (Da Silva *et al.,* 2002; Fischer *et al.,* 2004; Costa *et al.,* 2013). 

Overall, the micro- and macrostructural profiles presented by grouper species indicate an intermediate evolutionary rate between clades with larger (Silva *et al.,* 2020) and much lower (Getlekha *et al.,* 2016b) degrees of chromosomal variation.

### Historical cytobiogeography and karyotype divergences

The Atlantic Ocean represents the probable origin center of the Epinephelidae family, from where lineages moved from its eastern region and colonized the Indian and Pacific Oceans by the Tethys Sea (Ma *et al.,* 2016). During their extensive evolutionary history, estimated at 60 Mya (Ma *et al.,* 2016), groupers experienced an extraordinary conservation of the diploid number (2n = 48; all currently analyzed species), followed by a less extensive conservatism of the chromosomal morphologies (~60% of species). Notably, the enlarged set of the karyotype patterns of the groupers, including the eight species investigated here, evidenced an increase in the karyotype diversification associated to the historical-geographic dispersion of their species. Indeed, while in the Atlantic Ocean, 87% of the analyzed species share the 2n = 48a basal karyotype (Table 2), this pattern is reduced to 56% of the Pacific, 55% of the Indo-Pacific, and only to 33% of the Indian Ocean species (Figure 6).

Until the Miocene, approximately 23 Mya, epinephelids had a low diversity in the Indian and Pacific oceans (Wilson and Rosen, 1998; Renema *et al.,* 2008). When the invasion of the Indo-Pacific region occurred, historical tectonic processes promoted multiple reef habitats in that region, generating conditions for distinct evolutionary opportunities (Rohde and Muller, 2005; Carpenter *et al.,* 2011). Indeed, sympatric and allopatric divergences in a short period of time, defined the contemporary diversity of the groupers (Craig *et al.,* 2001; Ma *et al.,* 2016; Ma and Craig, 2018), in agreement with the karyotype diversification of some groups. 

Some features such as hermaphroditism, reproductive aggregations, high dispersive potential, and ecological plasticity are considered as gene flow maintainers and contributors to karyotype stability among groupers, as well as physical environment characteristics (Molina *et al.,* 2014; Motta-Neto *et al.,* 2019). In this case, the exploration and historical adaptation to new habitats may have had a disturbing effect on the modern grouper lineages, contributing to the disruption of the latent stability of the karyotype in the new colonization areas. Consequently, changes in the genome related to transposable elements (Schrader and Schmitz, 2019) and other repetitive sequences were established. In this context, adaptive pericentric inversions (Hoffmann and Rieseberg, 2008) could also be fixed as derived traits in some Epinephelidae species. 

Notably, cytogenetic patterns of serranids have maintained a basal karyotype with 2n = 48 chromosomes for a long period since their origin. Chromosomal homeologies are also evidenced by similar physical and compositional patterns of repetitive sequences such as ribosomal DNA, microsatellites, and transposable elements. Despite this, evident divergences in the evolution of the karyotype also occur, especially among the more recent Epinephelidae lineages, suggesting a close correlation with the colonization of new habitats and evolutionary circumstances. In fact, the set of chromosomal data available showed a more extensive karyotype diversification associated with geographic expansion events (Ma *et al.,* 2016) in the family. Therefore, the chromosomal evolution of the Epinephelidae proves to be more dynamic and diverse than supposed, with direct mediation of its historical and geographical contingencies.
